# A new species of *Chrysosplenium* (Saxifragaceae) from Shaanxi, north-western China

**DOI:** 10.3897/phytokeys.159.56109

**Published:** 2020-09-04

**Authors:** Long-Fei Fu, Rui Liao, De-Qing Lan, Fang Wen, Hong Liu

**Affiliations:** 1 Guangxi Key Laboratory of Plant Conservation and Restoration Ecology in Karst Terrain, Guangxi Institute of Botany, Guangxi Zhuang Autonomous Region and Chinese Academy of Sciences, Guilin 541006, China Guangxi Institute of Botany Guilin China; 2 College of Life Sciences & Key Laboratory for Protection and Application of Special Plant Germplasm in Wuling Area of Hubei Province, South-Central University for Nationalities, Wuhan 430074, Hubei Province, China South-Central University for Nationalities Wuhan China

**Keywords:** *Chrysosplenium zhouzhiense*, Sect. *Nephrophylloides*, Ser. *Macrophylla*, Subgen. *Gamosplenium*, taxonomy

## Abstract

*Chrysosplenium zhouzhiense* Hong Liu, a new species from Shaanxi, north-western China, is described and photographed. The new species belongs to Subgen. Gamosplenium Sect. Nephrophylloides Ser. Macrophylla and is most similar to *C. macrophyllum* and *C. zhangjiajieense* from which it differs by having a shorter stem, rhizome absent, basal leaf absent, sterile branch arising from the flowering stem and a light yellow flower with longer stamen. A global conservation assessment is performed and classifies *C. zhouzhiense* as Endangered (EN).

## Introduction

*Chrysosplenium* L. (Saxifragaceae) is a small perennial herbaceous genus that comprises ca. 70 species (Kim et al. 2019). *Chrysosplenium* is distributed throughout the temperate regions of the Northern Hemisphere, with high species diversity in eastern Asia, America and Europe (Hara 1957; Pan 1986a, b, 1992; Pan and Ohba 2001; Soltis 2007). China is one of the diversification centres of this genus including ca. 37 species, the majority of which are distributed in south-western, northern and central China, particularly the Provinces of Yunnan, Xizang, Sichuan and Shaanxi (Pan and Ohba 2001; Liu et al. 2016; Kim et al. 2019).

Based on the morphological features of leaf arrangement, *Chrysosplenium* was classified into two groups, namely, Sect. Alternifolia Franch. and Sect. Oppositifolia Franch. (Franchet 1890–1891). However, Hara (1957) argued that this classification was not natural due to the characters of flower, capsule and seed being highly variable within each section. He proposed a classification of 17 series instead. Briefly, characters such as leaf arrangement, pedicel length, sterile branch position, capsule shape, seed surface, stem surface, ovary position, stamen length, leaf surface, leaf isomery, sepal length and basal leaf size are used to establish and distinguish the series (Hara 1957). Of these, leaf arrangement occurs as the primary character in the key to the series in his classification (Hara 1957). This character was also considered to establish subgenus by Pan (1986a, b) when he made the taxonomic revision of Chinese *Chrysosplenium*. Seed surface was also used as an important character to delimit sections in his classification (Pan 1986a, b). Using *matK* sequence data, Soltis et al. (2001) conducted the extensive phylogenetic study demonstrating that two sections/subgenera are both monophyletic and form two sister clades. Thus, they agreed with leaf arrangement as a good indicator of the relationship within the genus.

In 2019, we found an unknown species of non-flowering *Chrysosplenium* when conducting a field investigation in Shaanxi, north-western China. We revisited the same locality in 2020 and collected specimens with flowers. Morphologically, this unknown species belongs to Chrysosplenium Subgen. Gamosplenium Maxim., Sect. Nephrophylloides Turcz., Ser. Macrophylla by leaves all alternate, seed minute papillae, ovary semi-inferior and disc absent (Pan 1986a, b). A thorough literature survey (Pan 1992; Pan and Ohba 2001; Liu et al. 2016; Kim et al. 2019) and review of herbarium specimens at A, E, HNWP, HZU, IBSC, KUN, MO, NEFI, NWTC, PE, SI, WUK (herbarium acronyms according to Index Herbariorum; Thiers 2019), suggested that it is a distinct and undescribed species.

## Materials and methods

### Morphology examination and conservation assessments

Photographs of the plant habit and morphological characters were taken in the field. All available specimens of the new species were deposited at the herbarium of South-Central University for Nationalities (**HSN**) and the herbarium of Guangxi Institute of Botany (**IBK**). All morphological characters from three specimens were studied using a dissecting microscope (SMZ171, Motic, China). For seed morphology, we also undertook SEM observation; seed materials were collected from the field and dried by silica gel. Seeds were placed in a bath-type ultrasonic cleaner for 10 min with 70% ethanol to remove impurities. After air-drying, the seeds were mounted using double-sided adhesive tape and coated with gold in a sputter coater, then observed and photographed under a Hitachi SU8010 scanning electron microscope. At least ten seeds were used to determine the size and surface. Conservation assessment was undertaken following IUCN (2019).

### Distribution map

A distribution map of *Chrysosplenium zhouzhiense*, *C. macrophyllum* Oliv. and *C. zhangjiajieense* X.L.Yu, Hui Zhou & D.S.Zhou was made using the software ArcGIS 10.2 (ESRI, Inc.). The geographical information for three species was obtained from the Global Biodiversity Information Facility (GBIF, https://www.gbif.org/zh/), and Chinese Virtual Herbarium (CVH, http://www.cvh.ac.cn/) and specimens deposited at HSN. We retained one accession per County to display the geographical range of each species. Specimens with ambiguous or incorrect identification were not used in this study.

## Taxonomic treatment

### Chrysosplenium zhouzhiense

Taxon classificationPlantaeSaxifragalesSaxifragaceae

Hong Liu sp. nov.

4CA98531-2EEE-565A-AFFA-EDA3F03B167B

urn:lsid:ipni.org:names:77211388-1

Figs 1 , 2 , 3 , 4

#### Diagnosis.

Most similar to *Chrysosplenium macrophyllum* and *C. zhangjiajieense* from which it differs by having a shorter stem, rhizome absent, basal leaf absent, sterile branch arising from flowering stem, light yellow flower with longer stamen.

**Figure 1.**
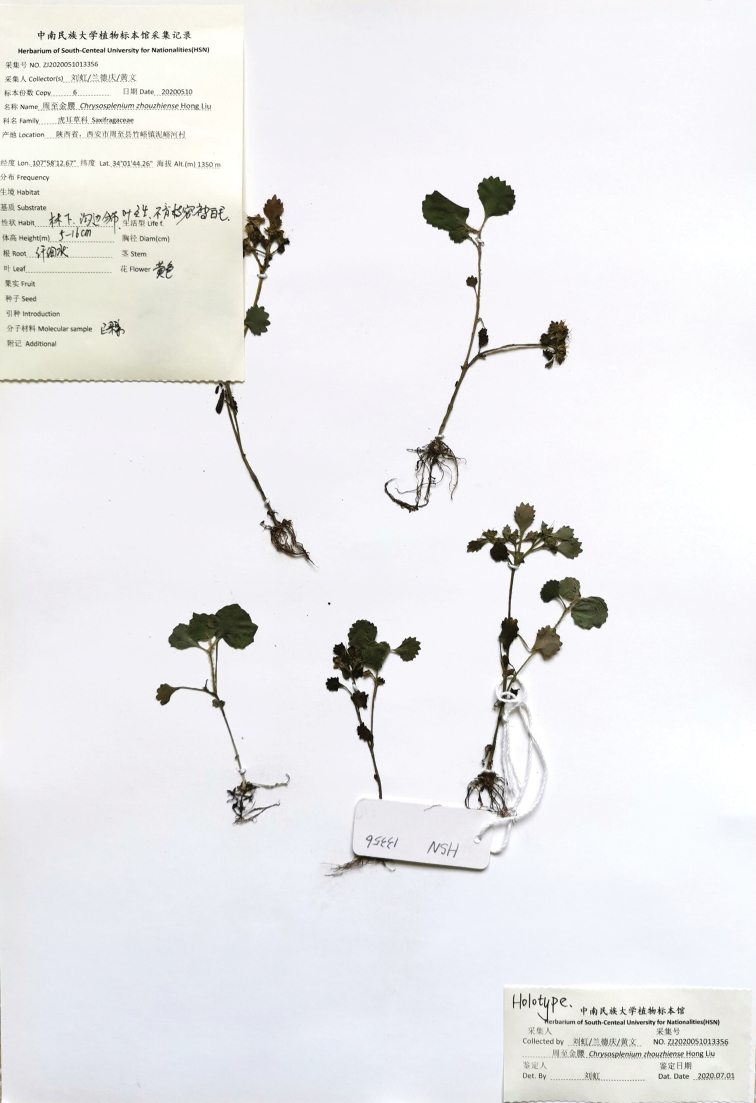
Type specimen of *Chrysosplenium zhouzhiense* Hong Liu, sp. nov. (Photo by Hong Liu).

#### Type.

China. Shaanxi: Niguhe Village, Zhouzhi County, Xi’an City, 34°01'44"N, 107°58'12"E, under broad-leaved forests in a mountain area at ca. 1350 m altitude, 10 May 2020, *Hong Liu, De-Qing Lan and Wen Huang HSN13356* (holotype HSN; isotypes HSN, IBK).

**Figure 2.**
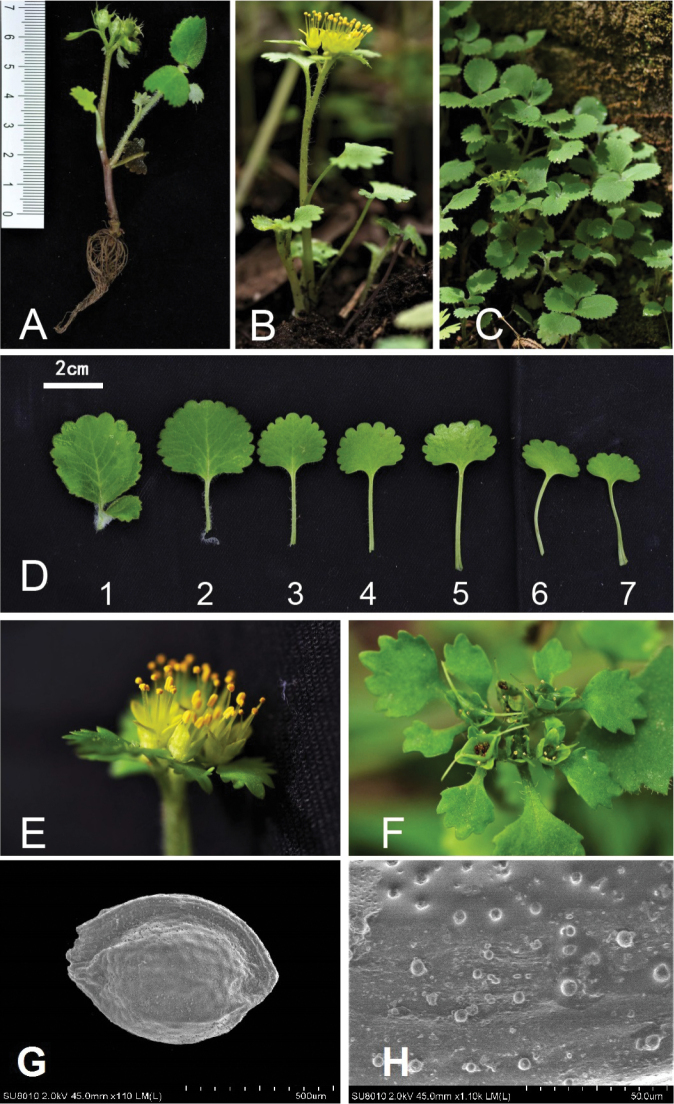
*Chrysosplenium zhouzhiense* Hong Liu, sp. nov. **A** fruiting plant **B** flowering plant **C** habitat **D** leaves of sterile branch **E** flowers close-up view **F** capsules **G, H** seeds, scanning electron micrograph, 110× (**G**) and 1,100× (**H**). (Photos by Hong Liu).

#### Description.

Perennial herbs, 5–16 cm high. Root fibrous and soft. Stolons 1–3, filiform, without long creeping rhizome or bulbs. Flowering stem(s) erect, simple, 5–15 cm high, smooth and subglabrous at base, dark red, rounded. Sterile branch 1 or (2), arising from the lower part and 2–7 cm above the base of flowering stem, rounded, 5–13 cm long, upper-middle part densely covered with white villose, hairs ca. 2–3 mm long. Basal leaves absent. Cauline leaves of flowering stem 2–4, alternate, slender, petiole 10–25 mm long; blade 5–12 × 7–15 mm, flabelliform or subrounded, sparsely white villose or subglabous, apex rounded, margin obtusely dentate (10–13 teeth), base cuneate to subcordate. Leaves of sterile branches 4–8, alternate, heterophyllous, upper leaves 3–4 crowded at stem apex larger, petiole 5–15 mm long, covered with soft downy hairs; blade 10–30 × 10–30 mm, flabelliform, densely lanate at both surfaces, apex subtruncate to rounded, margin undulate-crenate (12–16 teeth), base truncate to round; lower leaves 2–4, petiole 8–25 mm long; blade 5–15 × 5–15 mm, flabelliform, sparsely pubescent or subglabrous at both surfaces, apex subtruncate to rounded, margin undulate-crenate (8–11 teeth), base decurrent. Inflorescence often 6-flowered cyme, dense, 2–5 cm wide, branches glabrous or sparsely pubescence, surrounded by leaf-like bracts; bracteal leaves green, broadly ovate or obovate, rarely rounded, smooth at both surfaces, margin or petioles sparsely villose, base slightly oblique, broadly cuneate, triangular and two-rounded arranged, unequal; middle one major, petiole 5–8 mm long, blade 5–12 × 4–12 mm, margin obtusely dentate (7–11 teeth); two lateral ones minor, petiole 2–5 mm long, blade 3–5 × 2–4 mm, margin obtusely dentate (3–5 teeth). Flowers tetramerous, actinomorphic; sepals 4 (2 pairs), erect, yellow in flowering time, but turn green in fruiting time, 2.6–3.9 × 1.8–2.2 mm, ovate, apex acuminate; stamens 8, homostylic, 6–8 mm long, twice longer than sepals; filaments slender, 6–7 mm long; anther yellow, 2-locular, longitudinally dehiscent; ovary 2-locular, semi-inferior; stigma 2, 3–4 mm long; styles erect, shorter than stamens, 2–3 mm long. Fruit a capsule, 3–4 mm long, green, smooth, 2-lobed (horn-shaped), equal, dehiscent along the adaxial suture; seeds numerous, dark brown, ovoid, a raphe on one side, 550–640 × 350–450 μm, minute papillae.

**Figure 3.**
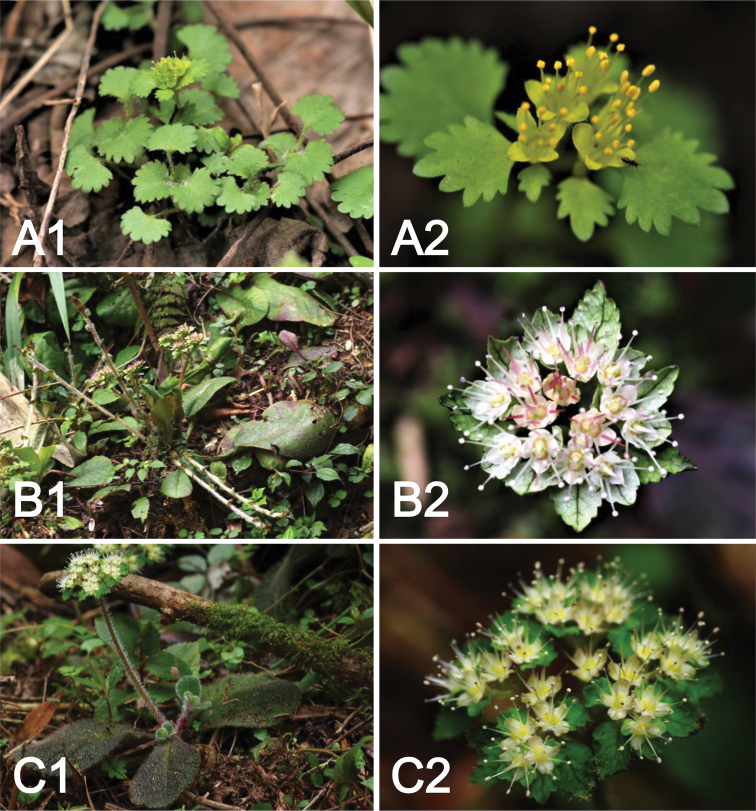
*Chrysosplenium* spp. habit and inflorescence **A***C. zhouzhiense* Hong Liu, sp. nov., habit (**A1**), inflorescence with yellow flower (**A2**) **B***C. macrophyllum*, habit (**B1**), inflorescence with white flower (**B2**) **C***C. zhangjiajieense*, habit (**C1**), inflorescence with white flower (**C2**).

#### Etymology.

*Chrysosplenium zhouzhiense* is named after the type locality, Zhouzhi County, Shaanxi Province, China.

#### Vernacular name.

zhōu zhì jīn yāo (Chinese pronunciation); 周至金腰 (Chinese name).

**Figure 4.**
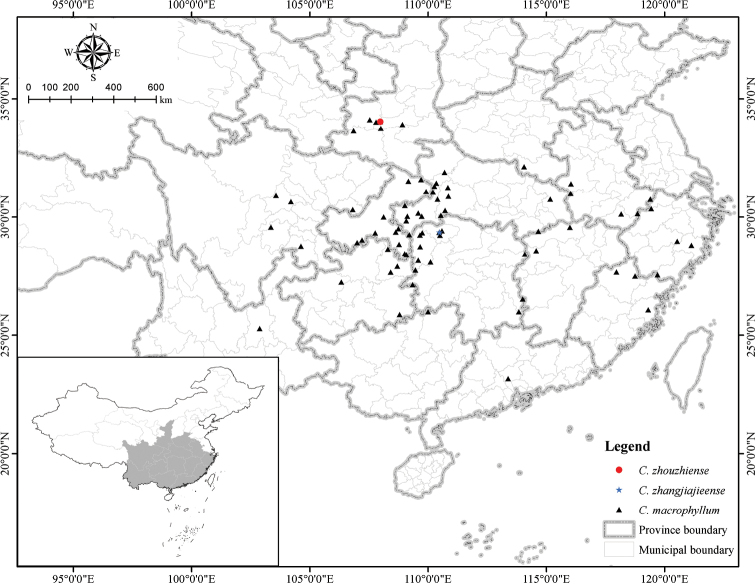
Distribution map of *Chrysosplenium zhouzhiense* (red circle), *C. macrophyllum* (black triangle) and *C. zhangjiajieense* (blue star).

#### Discussion.

*Chrysosplenium zhouzhiense* is characterised by leaves all alternate, seed minute papillae, ovary semi-inferior and disc absent. Thus, it belongs to Chrysosplenium Subgen. Gamosplenium, Sect. Nephrophylloides, Ser. Macrophylla (Pan 1986a, b). Chrysosplenium Ser. Macrophylla contains five species including *C. chinense* (Hara) J.T.Pan, *C. davidianum* Dence. ex Maxim, *C. macrophyllum* Oliv., *C. glossophyllum* Hara and a recently-described species *C. zhangjiajieense* (Pan 1986a; Liu et al. 2016). Amongst them, the new species is most similar to *C. macrophyllum* and *C. zhangjiajieense* by stamens long exserted from sepals, but can be distinguished based on plant height, rhizome, stolon, sterile branch, basal leaf, flower colour and stamen morphology as summarised in Table 1.

**Table 1.** Morphological comparison of *Chrysosplenium zhouzhiense*, *C. macrophyllum* and *C. zhangjiajieense*.

Characters	*C. zhouzhiense*	*C. macrophyllum*	*C. zhangjiajieense*
Plant height	5–16 cm	17–21 cm	13–19 cm
Rhizome	absent	thick	thick
Stolon	1–3	absent	absent
Flowering stem and sterile branch	sterile branch arising from flowering stem	separate	separate
Cauline leaf	2–4	1	1
Basal leaf	absent	large, 2.3–19 cm long	large, 4–10.5 cm long
Flower colour	light yellow	white	white
Stamen	6–8 mm	4–6 mm	4–6 mm

#### Conservation Status.

At present, *Chrysosplenium zhouzhiense* is only known from a single locality (IUCN 2019). At this locality, the population of this species comprises ca. 100 mature individuals (< 250 mature individuals, criteria D1). Therefore, we propose that *C. zhouzhiense* should be considered as Endangered D1 (EN) according to IUCN criteria (IUCN 2019).

##### Key to species of Chrysosplenium Subgen. Gamosplenium Sect. Nephrophylloides Ser. Macrophylla modified from Pan and Ohba (2001)

**Table:** 

1	Stamens long exserted from sepals	**2**
–	Stamens subequalling or shorter than sepals	**3**
2	Stolon 1–3; basal leaf absent; flower light yellow; stamen 6–8 mm	***C. zhouzhiense***
–	Stolon absent; basal leaf large; flower white; stamen 4–6 mm	**4**
3	Stem glabrous; basal leaves reniform to orbicular-reniform	***C. chinense***
–	Stem brown villous; basal leaves broadly ovate to orbicular	**5**
4	Basal leaves sparsely pilose; cauline leaves 12–17 × 5–7.5 mm, narrowly elliptic	***C. macrophylla***
–	Basal leaves densely long villous; cauline leaves 3–5 × 4–6 mm, oval to circular	***C. zhangjiajieense***
5	Sterile branches absent; basal leaf margin 20–36-crenate, sometimes obscurely so; stamens shorter than sepals; capsule rostrums ca. 0.5 mm	***C. glossophyllum***
–	Sterile branches arising from stem base; basal leaf margin (7–)13–17-crenate; stamens subequalling sepals; capsule rostrums 1–2 mm	***C. davidianum***

## Supplementary Material

XML Treatment for Chrysosplenium zhouzhiense
